# SARS-CoV-2 Spike Protein-Directed Monoclonal Antibodies May Ameliorate COVID-19 Complications in APECED Patients

**DOI:** 10.3389/fimmu.2021.720205

**Published:** 2021-08-24

**Authors:** Elise M. N. Ferré, Monica M. Schmitt, Sebastian Ochoa, Lindsey B. Rosen, Elana R. Shaw, Peter D. Burbelo, Jennifer L. Stoddard, Shakuntala Rampertaap, Tom DiMaggio, Jenna R. E. Bergerson, Sergio D. Rosenzweig, Luigi D. Notarangelo, Steven M. Holland, Michail S. Lionakis

**Affiliations:** ^1^Fungal Pathogenesis Section, LCIM, NIAID, NIH, Bethesda, MD, United States; ^2^Immunopathogenesis Section, LCIM, NIAID, NIH, Bethesda, MD, United States; ^3^Human Immunological Diseases Section, LCIM, NIAID, NIH, Bethesda, MD, United States; ^4^National Institute of Dental and Craniofacial Research, NIH, Bethesda, MD, United States; ^5^Immunology Service, Department of Laboratory Medicine, NIH Clinical Center, NIH, Bethesda, MD, United States; ^6^Immune Deficiency Genetics Section, LCIM, NIAID, NIH, Bethesda, MD, United States

**Keywords:** APECED, APS-1, AIRE, type-1 IFN autoantibodies, pneumonitis, COVID-19, bamlanivimab-etesevimab

## Abstract

Patients with the monogenic immune dysregulatory syndrome autoimmune polyendocrinopathy-candidiasis-ectodermal dystrophy (APECED), which is caused by loss-of-function mutations in the autoimmune regulator (*AIRE*) gene, uniformly carry neutralizing autoantibodies directed against type-I interferons (IFNs) and many develop autoimmune pneumonitis, both of which place them at high risk for life-threatening COVID-19 pneumonia. Bamlanivimab and etesevimab are monoclonal antibodies (mAbs) that target the SARS-CoV-2 spike protein and block entry of SARS-CoV-2 in host cells. The use of bamlanivimab and etesevimab early during infection was associated with reduced COVID-19–associated hospitalization and death in patients at high risk for progressing to severe disease, which led the US Food and Drug Administration to issue an emergency use authorization for their administration in non-hypoxemic, non-hospitalized high-risk patients. However, the safety and efficacy of these mAbs has not been evaluated in APECED patients. We enrolled two siblings with APECED on an IRB-approved protocol (NCT01386437) and admitted them prophylactically at the NIH Clinical Center for evaluation of mild-to-moderate COVID-19. We assessed the safety and clinical effects of early treatment with bamlanivimab and etesevimab. The administration of bamlanivimab and etesevimab was well tolerated and was associated with amelioration of COVID-19 symptoms and prevention of invasive ventilatory support, admission to the intensive care, and death in both patients without affecting the production of antibodies to the nucleocapsid protein of SARS-CoV-2. If given early in the course of COVID-19 infection, bamlanivimab and etesevimab may be beneficial in APECED and other high-risk patients with neutralizing autoantibodies directed against type-I IFNs.

## Introduction

Studies seeking to identify risk factors for severe COVID-19 uncovered genetic and acquired defects in type-I IFN pathways in a proportion of patients with life-threatening COVID-19 pneumonia. In a cohort of such patients, ~3% of individuals harbored loss-of-function mutations in genes involved in type-I IFN signaling (1). TLR7 variants that were associated with impaired type-I IFN responses were independently reported in a few patients with severe COVID-19 (2). A separate study showed that ~10% of individuals with severe COVID-19 pneumonia had neutralizing autoantibodies directed to type-I IFNs (3). The presence of these autoantibodies in patients with critical COVID-19 pneumonia was independently verified in other patient cohorts (4, 5) and was recently shown to be associated with delayed viral clearance during COVID-19 infection (6), underscoring the importance of type-I IFNs in the protective immune response against SARS-CoV-2 (7)

Autoantibodies directed to type-I IFNs –predominantly to IFN-α and IFN-ω, and infrequently to IFN-β– are a characteristic and almost universal feature of APECED or autoimmune polyglandular syndrome type-1 (APS-1) (8), a monogenic disorder resulting from loss-of-function *AIRE* mutations, which impair central immune tolerance (9–12). We recently reported high rates of COVID-19–associated hospitalization, intensive care unit (ICU) admission, mechanical ventilation, and death in 22 APECED patients with COVID-19; these patients carried type-I IFN-targeted neutralizing autoantibodies (3, 13, 14). Therapeutic approaches aimed to boost viral clearance and type-I responses have been described in some APECED and other patients with type-I IFN autoantibodies, including administration of IFN-β, remdesivir, and/or plasmapheresis (13, 15–17).

However, there are currently limited therapies for patients with a high risk of progression to severe COVID-19. Bamlanivimab and etesevimab are monoclonal antibodies (mAbs) that bind to the spike protein of SARS-CoV-2 thereby blocking entry into host cells (18, 19). Bamlanivimab and etesevimab bind to distinct yet overlapping epitopes within the receptor binding domain (RBD) of SARS-CoV-2 spike protein (**Supplementary Figure 1**). Banlanivimab is able to bind RBD in its open and closed conformations, while etesevimab binds only to the open conformation (18, 19). In the open conformation, the RBD domain is exposed and able to bind ACE2, while in the closed conformation, it is occluded. Because RBD fluctuates between its open and closed conformations, targeting both allows for greater neutralization. Together, both antibodies block a greater area of attachment between the spike protein of SARS-CoV-2 and the ACE2 receptor. While the use of bamlanivimab alone is not recommended in the US due to an increase in resistant variants (20), a phase 2/3 clinical trial evaluating the combination of bamlanivimab and etesevimab showed a reduction in hospitalization risk when used in the outpatient setting, with the greater risk reduction observed in patients older than 65 years or with obesity (body mass index [BMI] ≥35 kg/m²) (21). More recently, another phase 2/3 clinical trial found that administration of bamlanivimab and etesevimab led to lower incidence of COVID-19-related hospitalizations and death in high-risk ambulatory patients (22). Therefore, the US Food and Drug Administration has issued an Emergency Use Authorization for these mAbs for the treatment of non-hospitalized, non-hypoxemic patients with mild-to-moderate COVID-19 who are at high risk for progressing to severe disease (21). Although APECED patients are at high risk for progression to severe COVID-19 pneumonia (13), the use of mAbs directed to the SARS-CoV-2 spike protein has not been studied in this population to date. Here we report the use of bamlanivimab and etesevimab in two APECED patients with neutralizing type-I IFN autoantibodies and pre-existing lung disease who were infected with SARS-CoV-2.

## Materials and Methods

Both patients were enrolled on an IRB-approved protocol (NCT01386437) (23) and admitted to the NIH Clinical Center for evaluation and management of COVID-19. The two patients were not included in our recent report (13). Median inhibitory concentration (IC50) curves representing IFN-α2– and IFN-ω–induced pSTAT1 levels were determined by assessing STAT1 phosphorylation [Alex Fluor 647 mouse anti-Stat1 (pY701), BD Biosciences cat# 612597] by flow cytometry in healthy donor CD14+ monocytes (FITC anti-human CD14, BD Biosciences, cat#555397) after 15 minutes of stimulation with 10 ng/mL of IFN-α2 (R&D, cat# 11101-2) or 10 ng/mL of IFN-ω (Peprotech, cat# 300-02J) in the presence of 10% human serum AB (Innovative Research, cat# IPLA-SERAB-OTC-HI) or patient plasma serially diluted into human serum AB. The stimulation index (stimulated over unstimulated condition) for serial dilutions of patient plasma was normalized against that of 10% human serum AB. Data were analyzed using FlowJo version 10 with graphs made in GraphPad Prism 8.

Luciferase immunoprecipitation systems (LIPS) assays were used for measuring antibodies against the SARS-CoV-2 nucleocapsid and spike proteins as previously described (24). For these measurements, standardized LIPS assays for both viral antigens with established cut-off values were utilized. Using a microtiter plate format, protein A/G beads were used to capture the antibody-antigen complexes and after extensive washing of the complexes, luciferase activity was quantified as light units (LUs) in a luminometer using coelenterazine as a substrate. All LU data presented was obtained from the average of two determinations.

Immunophenotyping of immune cells was performed as previously described (23, 25). Frozen peripheral blood mononuclear cells (PBMCs) were thawed and stained using the following fluorochrome-conjugated (FITC, PE, PE-Cy7, APC, APC-eFluor 780, Alexa Fluor 700, eFluor 450, PerCP-Cy5.5) antibodies against human CD45 (HI30), CD19 (SJ25C1), CD27 (TNFRSF7), CD10 (CB-CALLA) (eBioscience); CD3 (SK7), CD16 (SK7), CD56 (SK7), CD20 (L27), CD62L (SK11), CD38 (HB7) (BD Biosciences); IgM (MHM-88), CD21 (Bu32) (BioLegend); CD4 (S3.5), CD8 (3B5) (ThermoFisher Scientific); CD45RA (ALB11) (Beckman Coulter) for 30 minutes on ice. After incubation, the cells were washed with FACS buffer and the samples acquired using a FACSCanto (BD Biosciences). FlowJo (TreeStar) was used for the final analysis. Cell numbers were quantified from the FACS plot percentages using the patient’s same-day absolute lymphocyte count obtained from the complete blood count (CBC) with differential. Absolute monocyte and neutrophil counts were obtained from the patients’ CBC with differential.

## Results

**Figure 1:**
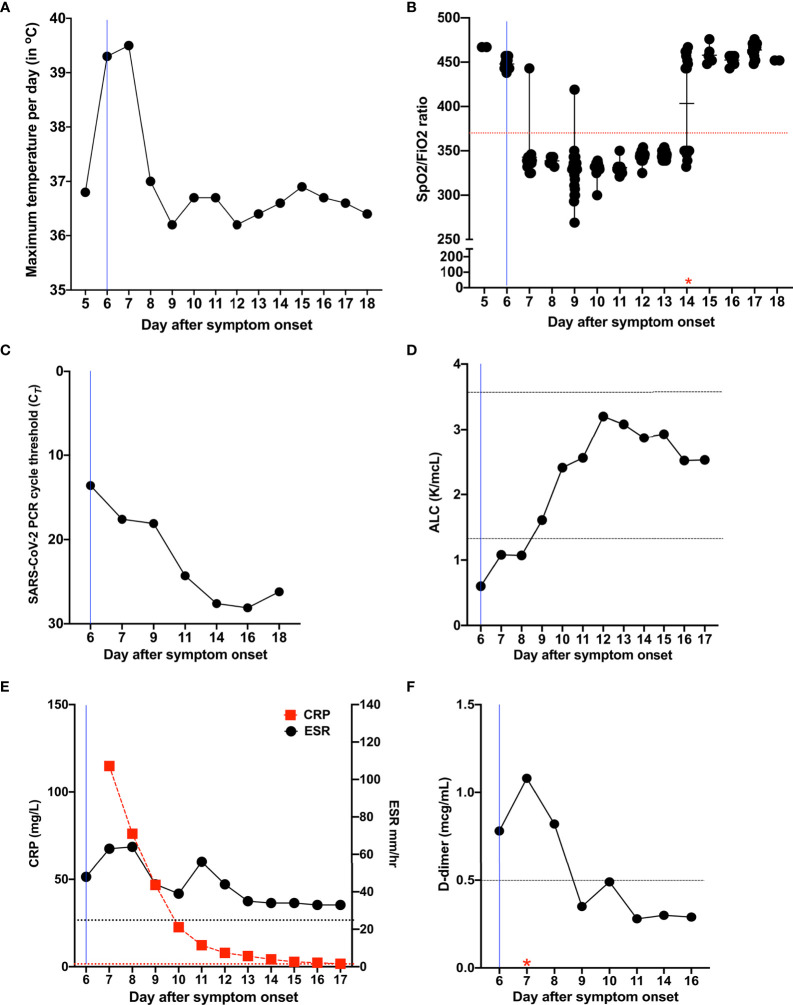
Temporal evolution of clinical and laboratory characteristics in Patient 1 during the course of COVID-19. **(A)** Temporal evolution of maximum temperature per day in degrees Celsius (°C). **(B)** Measures of oxygen uptake requirements SpO2/FiO2 [% blood oxygen saturation (SpO2)/fraction of delivered oxygen (FiO2)], a ratio that accounts for both oxygen delivery and uptake (maximum, 476 for 100% oxygen saturation on room air). The red star indicates the day of discontinuation of supplemental oxygen. **(C)** Temporal evolution of cycle threshold (Ct) of SARS-CoV-2 PCR test results. **(D)** Temporal evolution of absolute lymphocyte count (normal range, 1.37 to 3.57 K/µl). **(E)** Temporal evolution of the inflammatory markers CRP (red; normal range, < 3 mg/L) and ESR (black; normal range, < 25.0 mm/hr). **(F)** Temporal evolution of D-dimer (normal range, < 0.5 µg/mL). The red star indicates the day of initiation of anticoagulation. The blue line in all figures corresponds to the day when the patient was initiated on methylprednisolone, remdesivir, and bamlanivimab and etesevimab. Horizontal dotted lines indicate normal range. ALC, absolute lymphocyte count; CRP, C-reactive protein; ESR, erythrocyte sedimentation rate.

**Figure 2:**
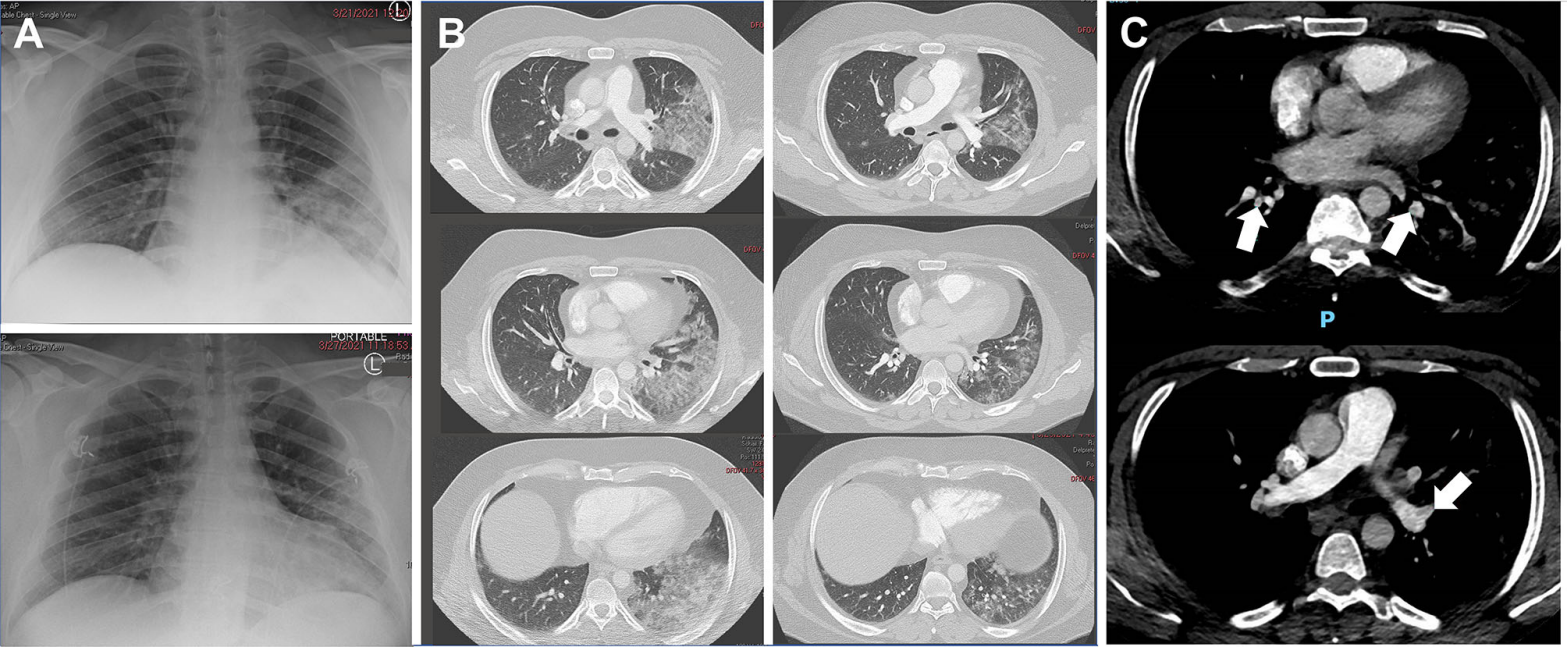
Temporal evolution of radiographic abnormalities in Patient 1 during the course of COVID-19. **(A)** Chest X-ray demonstrating left lower lung airspace disease on admission at the NIH Clinical Center (upper panel) and radiographic improvement noted 7 days later (lower panel). **(B)** Chest CT showing left>right pulmonary infiltrates on admission at the NIH Clinical Center (Left panels) and radiographic improvement noted 7 days later (right panels). **(C)** Chest CT angiography on admission at the NIH Clinical Center showing small pulmonary emboli (white arrows).

Patient 1 is a 37-year-old obese male (BMI, 42) with APECED (homozygous for c.967_979del13 in *AIRE*) and neutralizing autoantibodies directed against IFN-α2 and IFN-ω but not IFN-β (**Supplementary Figures 2A, B**). His APECED manifestations include chronic mucocutaneous candidiasis (CMC), adrenal insufficiency, nail dystrophy, alopecia, hypothyroidism, enamel hypoplasia, gastritis, and pneumonitis with associated bronchiectasis (**Table 1**), which develops in up to ~40% of patients (25, 26). His medications at the time of COVID-19 diagnosis are summarized in **Table 1**. He developed fever, dry cough, exertional dyspnea, and headache four days after his children became symptomatic with COVID-19. He tested positive for SARS-CoV-2 by PCR two days after symptom onset. Given the high risk for progression to severe COVID-19, we prophylactically admitted him to the NIH Clinical Center on day 5 after symptom onset. Upon admission, he was afebrile while receiving anti-pyretic therapy, and his oxygen saturation was normal (SpO2, 98%) (**Figures 1A, B**). A nasopharyngeal swab SARS-CoV-2 PCR test was positive with a cycle threshold (Ct) value of 13.6 indicative of a high viral load (**Figure 1C**). Laboratory studies were significant for a decreased absolute lymphocyte count (ALC) and elevated C-reactive protein (CRP), erythrocyte sedimentation rate (ESR), D-dimer (**Figures 1D–F**), absolute neutrophil count (ANC) (7.74 K/µL), absolute monocyte count (AMC) (0.91 K/µL) (**Supplementary Figure 3A**), lactate dehydrogenase (LDH) (260 units/L), and fibrinogen (491 mg/dL), while ferritin was normal (83 mg/L). A chest X-ray (CXR) showed left lower lung airspace disease (**Figure 2A**) and a chest computed tomography (CT) angiography revealed bilateral (left>right) ground-glass opacities (**Figure 2B**) and multiple pulmonary artery filling defects consistent with small pulmonary emboli (**Figure 2C**). On day 6 after symptom onset, he received bamlanivimab and etesevimab over a 2-hour infusion with cetirizine and acetaminophen pre-treatment and tolerated it well. Given the high SARS-CoV-2 viral load, we opted to initiate remdesivir. He also received doxycycline and ceftriaxone for community-acquired pneumonia coverage, and enoxaparin for pulmonary emboli.

**Table 1:** Clinical manifestations and medications of the APECED patients included in our study.

	Patient 1	Patient 2
**Clinical manifestations**		
APECED manifestations	CMC, AI, ND, alopecia, HT, gastritis, pneumonitis, EH	CMC, HP, AI, gastritis, IM, EH, SS, vitiligo
Other diagnoses	Chronic sinusitis, hypogammaglobulinemia, ITP, migraines, seizure disorder, anxiety disorder, hyperlipidemia, sleep apnea	Asthma, CKD stage 3, hypogammaglobulinemia, chronic sinusitis, Grave’s disease with post-treatment HT, seizure disorder, bipolar disorder, migraines
**Medications at time of COVID-19 diagnosis**		
	Ascorbic acid	Cholecalciferol
	Atorvastatin	Fludrocortisone
	Fludrocortisone	Immune globulin
	Fluticasone nasal spray	Hydrocortisone
	Gabapentin	Levothyroxine
	Immune globulin	Magnesium oxide
	Hydrocortisone	Parathyroid hormone (recombinant)
	Levothyroxine	Oxcarbazepine
	Melatonin	Topiramate
	Mirtazapine	Zinc sulfate
	Omeprazole	
	Oxcarbazepine	
	Pseudoephedrine	
	Simethicone	
	Ubiquinone	
	Valacyclovir	
	Zinc sulfate	

CMC, chronic mucocutaneous candidiasis; HP, hypoparathyroidism; AI, adrenal insufficiency; ND, nail dystrophy; HT, hypothyroidism; IM, intestinal malabsorption; EH, enamel hypoplasia; SS, Sjogren’s-like syndrome; CKD, chronic kidney disease; ITP, idiopathic thrombocytopenia purpura.

On day 6 after symptom onset, he developed fever (Tmax 39.5°C) and became hypoxic with SpO2 of 91% (**Figures 1A, B**) and PaO2 of 63 mmHg necessitating supplemental oxygen (2 liters/min by nasal cannula). Because corticosteroids decrease COVID-19–associated hyper-inflammation and improve survival (27) and initiation of corticosteroids within 24 hours of hypoxemia onset was associated with improved outcomes in APECED patients (13), we initiated methylprednisolone (0.5 mg/Kg/day) on day 6 after symptom onset. Fever and cough resolved by day 8 after symptom onset. Although the patient remained hypoxic -primarily upon exertion- until day 14 after symptom onset at which point supplemental oxygen was discontinued, he did not require ICU care or escalation of oxygenation support to non-invasive or invasive ventilatory modalities. The SARS-CoV-2 viral load gradually declined with Ct values in the high-20 range at day 14 after symptom onset, eight days after infusion of bamlanivimab and etesevimab and following a 6-day course of remdesivir, which was discontinued on day 12 after symptom onset due to mildly elevated transaminases (ALT 63, AST 45) (**Figure 1C**). ALC, D-dimer, and CRP normalized by days 7, 9, and 15 after symptom onset, respectively (**Figures 1D–F**). Neutrophilia persisted during corticosteroid administration (**Supplementary Figure 3A**). CXR and chest CT demonstrated improvement of lung infiltrates at day 12 after symptom onset (**Figures 2A, B**). Prior to discharge on day 18 after symptom onset, he was transitioned to apixaban to complete a 3-month course and prednisone with a 4-week taper to prevent rebound inflammation.

**Figure 3:**
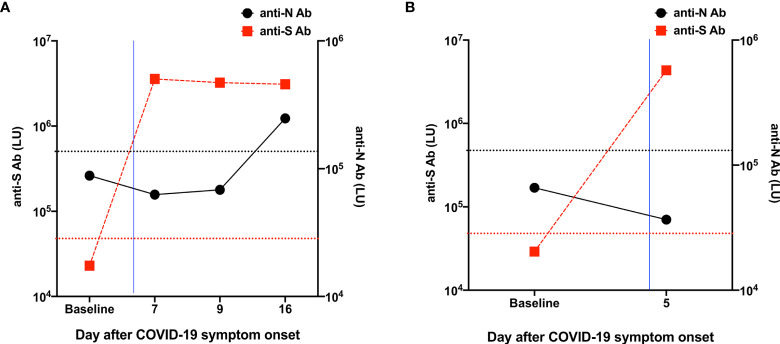
Measurement of anti-S and anti-N antibodies against SARS-CoV-2 in the patients included in our study. **(A)** The baseline value was obtained from a stored serum sample from the Patient 1 initial NIH visit in 2017. The blue line corresponds to the day when the patient received bamlanivimab and etesevimab. These findings demonstrate that the patient mounted a natural immune response to the nucleocapsid of SARS-CoV-2 despite the administration of bamlanivimab and etesevimab. **(B)** The baseline value was obtained from a stored serum sample from the Patient 2 initial NIH visit in 2017. The blue line corresponds to the day after the patient received bamlanivimab and etesevimab. Horizontal dotted red and black lines indicate cut-off thresholds of positivity for anti-S and anti-N antibodies, respectively, and were based on the mean plus 3 and 4 standard deviations (SDs), respectively, of the serum values derived from 32 uninfected blood donor controls.

Immunophenotyping of peripheral blood lymphocyte populations was performed at days 7, 9, and 16 after symptom onset (**Table 2**). The CD4/CD8 ratio was increased at all time points with decreased total CD8^+^ T cells at day 7 and increased total CD4^+^ T cells at days 9 and 16. Double negative T cells were decreased at day 7 and normalized at days 9 and 16. Effector CD4^+^ T cells were increased at days 7, 9, and 16 and effector memory CD4^+^ T cells were increased at days 9 and 16. Naïve and central memory CD4^+^ T and CD8^+^ T cells were decreased at days 7, 9, and 16. Effector memory CD8^+^ T cells were decreased at the pre-COVID-19 baseline and at day 7 after symptom onset and normalized at days 9 and 16. Total B cells were decreased at baseline, day 7, and day 9 and normalized at day 16. Among B cell subsets, the patient exhibited decreased frequencies of early transitional, transitional, and memory-switched cells after SARS-CoV-2 infection, consistent with pre-COVID-19 testing. He also had increased CD21^low^CD38^low^ B cells at days 7, 9, and 16, absent immature B cells, and decreased memory B cells at day 7 with normalization by day 9. Natural killer (NK) cells and natural killer T (NKT) cells were decreased at day 7 and normalized by day 9. We measured anti-S and anti-N antibodies to determine whether the administration of bamlanivimab and etesevimab affected the development of humoral responses (anti-N antibodies) to the SARS-CoV-2 infection. We found that the patient mounted an immune response to the SARS-CoV-2 nucleocapsid protein by day 16 after symptom onset and, as expected, we detected anti-S antibodies starting the day after administration of bamlanivimab and etesevimab (**Figure 3A**).

**Table 2:** Lymphocyte subsets before COVID-19 and during the course of COVID-19 infection in the APECED patients included in our study.

	Patient 1								Patient 2				Healthy Control	
	Baseline*		Day 7		Day 9		Day 16		Baseline*		Day 5		95% confidence interval	
	Percent	Number/μl	Percent	Number/μl	Percent	Number/μl	Percent	Number/μl	Percent	Number/μl	Percent	Number/μl	Percent	Number/μl
**CD3**	87.20%	1657	91.70%	981	90.10%	2171	85.90%	2173	96.40%	2150	89.60%	2115	55.3-87.7%	651-2804
**CD3/alpha beta**	83.30%	1583	89.40%	957	88.50%	2133	84.10%	2128	91.20%	2034	84.00%	1982	53.0-83.5%	585-2716
**CD3/gamma delta**	3.80%	72	2.30%	25	1.60%	39	1.80%	46	5.10%	114	5.60%	132	0.6-16.6%	11-288
**CD4/CD3**	66.90%	1271	78.80%	843	77.40%	**1865**	72.40%	**1832**	47.30%	1055	48.50%	1145	27.9-55.8%	370-1336
**CD8/CD3**	16.60%	315	10.20%	**109**	11.00%	265	11.00%	278	43.80%	977	36.10%	852	13.6-46.2%	185-1024
**CD4/CD8 ratio**	**4.03**	-	**7.73**	–	**7.04**	-	**6.58**	–	1.08	-	1.34	–	0.81-4.00	-
**CD3+/CD4-/CD8-**	3.50%	67	1.80%	**19**	1.60%	39	2.20%	56	4.00%	89	4.90%	116	1.2-11.5%	24-223
**CD4/CD3/CD62L+/CD45RA+ (Naïve)**	34.70%	659	4.40%	**47**	1.84%	**44**	1.05%	**27**	23.70%	529	7.28%	172	6.2-26.9%	87-796
**CD4/CD3/CD62L+/CD45RA- (Central memory)**	28.10%	534	5.19%	**55**	5.39%	**130**	3.77%	**95**	16.30%	363	7.32%	173	9.1-28.6%	166-544
**CD4/CD3/CD62L-/CD45RA- (Effector memory)**	4.10%	**78**	17.89%	191	26.24%	**632**	28.02%	**709**	6.10%	136	10.09%	238	3.7-12.9%	80-262
**CD4/CD3/CD62L-/CD45RA+ (Effector)**	0.00%	**0**	51.38%	**550**	43.89%	**1058**	39.53%	**1000**	1.20%	27	24.30%	**573**	0.1-5.5%	1-132
**CD8/CD3/CD62L+/CD45RA+ (Naïve)**	7.30%	139	0.20%	**2**	0.10%	**2**	0.10%	**3**	17.30%	386	4.70%	111	2.3-18.2%	37-484
**CD8/CD3/CD62L+/CD45RA- (Central memory)**	3.20%	61	0.10%	**1**	0.10%	**2**	0.20%	**5**	4.40%	98	1.00%	24	1.2-7.6%	19-175
**CD8/CD3/CD62L-/CD45RA- (Effector memory)**	1.90%	**36**	1.90%	**20**	3.40%	82	2.90%	73	5.20%	116	4.20%	99	2.0-12.4%	47-383
**CD8/CD3/CD62L-/CD45RA+ (Effector)**	4.20%	80	8.00%	86	7.40%	178	7.80%	197	16.90%	**377**	26.20%	**618**	0.9-13.6%	17-274
**CD20**	1.70%	**32**	2.50%	**27**	2.70%	**65**	5.40%	137	2.70%	**60**	9.00%	212	3.8-18.0%	79-399
**CD19**	1.70%	**32**	2.50%	**27**	2.70%	**65**	5.40%	137	2.70%	**60**	9.00%	212	3.8-18.0%	79-399
**CD20/CD27 (Memory)**	1.00%	19	1.10%	**12**	0.90%	22	2.50%	63	1.80%	40	7.00%	**165**	0.8-4.8%	18-120
**CD20/CD38 (Transitional)**	1.60%	**30**	0.00%	**0**	0.00%	**0**	0.00%	**0**	1.40%	**32**	1.20%	**28**	3.2-16.4%	65-358
**CD20/CD10 (Immature)**	0.30%	6	0.00%	**0**	0.00%	**0**	0.00%	**0**	0.10%	1	0.00%	**0**	0.1-2.6%	1-64
**CD21/CD10 (Immature)**	0.10%	2	0.00%	**0**	0.00%	**0**	0.00%	**0**	0.00%	**0**	0.00%	**0**	0.1-1.5%	1-32
**CD20/IgM-/CD38++ (Plasmablasts)**	0.00%	0	0.00%	0	0.00%	0	0.00%	0	0.00%	0	0.00%	0	0-0.1%	0-1
**CD20/IgM+/CD10+ (Immature)**	0.00%	**0**	0.00%	**0**	0.00%	**0**	0.00%	**0**	0.00%	**0**	0.00%	**0**	0.1-1.4%	1-30
**CD20/CD38+/CD10+ (Early transitional)**	0.00%	**0**	0.00%	**0**	0.00%	**0**	0.00%	**0**	0.00%	**0**	0.00%	**0**	0.1-1.4%	1-32
**CD20/CD27/IgM+ (Memory non-switched)**	0.60%	11	0.90%	10	0.80%	19	2.30%	58	0.00%	**0**	6.20%	**146**	0.3-2.5%	6-65
**CD20/CD27/IgM- (Memory switched)**	0.10%	**2**	0.10%	**1**	0.10%	**2**	0.40%	10	0.00%	**0**	0.10%	**2**	0.3-2.2%	6-49
**CD19/CD21 low/CD38 low (Autoreactive)**	0.30%	6	1.10%	**12**	1.00%	**24**	1.80%	**46**	0.10%	2	3.50%	**83**	0-0.4%	0-9
**CD16+orCD56+/CD3- (NK)**	10.80%	205	5.80%	**62**	6.80%	164	8.40%	213	1.50%	**33**	1.50%	**35**	7.3-33.4%	126-841
**CD16+orCD56+/CD3+ (NKT)**	9.00%	171	3.30%	**35**	3.10%	75	3.90%	99	13.90%	310	19.60%	**463**	2.1-18.0%	56-448

Absolute numbers highlighted in red are elevated above the reference range. Absolute numbers highlighted in blue are decreased below the reference range. Lymphocyte % reflect frequencies within total lymphocytes in blood.

*Baseline values before COVID-19 infection were obtained from fresh whole blood at a previous visit to NIH in 2017 (for Patient 1) and in 2020 (for Patient 2).

Patient 2 is the 39-year-old sister to Patient 1. She is also homozygous for c.967_979del13 in *AIRE* and carries neutralizing autoantibodies against IFN-α and IFN-ω but not IFN-β (**Supplementary Figures 2A, B**). Her APECED manifestations include CMC, hypoparathyroidism, adrenal insufficiency, gastritis, intestinal malabsorption, enamel hypoplasia, Sjogren’s-like syndrome, vitiligo, and she also suffered from asthma (**Table 1**). Her medications at the time of COVID-19 diagnosis are listed in **Table 1**. She first developed headache and altered taste and smell following exposure to her symptomatic brother before his NIH admission. She developed cough and dyspnea at rest on day 2 and pleuritic chest pain on day 3 after symptom onset, at which point she was prophylactically admitted to the NIH Clinical Center. A saliva SARS-CoV-2 PCR test was positive with a Ct value of 21.3. Admission vital signs (temperature, 36.6°C; SpO2, 98%), laboratory values (ALC, ANC, AMC, CRP, ESR, D-dimer, LDH, fibrinogen, ferritin) and imaging studies (CXR, chest CT) were normal (**Supplementary Figure 3B**). On day 4 after symptom onset, she received a 2-hour infusion of bamlanivimab and etesevimab with cetirizine and acetaminophen pre-treatment and tolerated it well. She had resolution of altered taste and smell, cough and dyspnea, and headache within 1, 2, and 3 days following bamlanivimab and etesevimab infusion, respectively. She was discharged on day 5 after symptom onset without further complications.

Immunophenotyping of peripheral blood lymphocyte populations performed on day 5 after symptom onset revealed increased frequencies of CD4^+^ and CD8^+^ effector cells and normal representation of other T cell subsets with normal CD4/CD8 ratio (**Table 2**). Among B cells, the frequencies of memory, memory non-switched, and CD21^low^CD38^low^ cells were increased. The frequencies of early transitional, transitional, immature, and memory switched B cell subsets were decreased, consistent with pre-COVID-19 testing. NK cells were decreased and NKT cells were increased. Evaluation of anti-S and anti-N antibodies on day 5 after symptom onset (**Figure 3B**), a day after infusion of bamlanivimab and etesevimab, showed expected positivity for anti-S antibodies but negative anti-N antibodies, which typically develop between 8 and 14 days after symptom onset (24). No patient samples were available later in the course of SARS-CoV-2 infection to determine whether and when anti-N antibodies were generated in response of the infection.

## Discussion

International cohorts of patients with inborn errors of immunity who developed COVID-19 reported higher rates of mechanical ventilation (16%) and death (10%) compared to the general population (28). In particular, patients with genetic defects in type-I IFN pathways or neutralizing autoantibodies directed against type-I IFNs, including APECED patients, are among those carrying a high risk for severe disease. A recent study describing the clinical course of 22 APECED patients with COVID-19 showed higher rates of ICU admission (68%), mechanical ventilation (50%) and death (18%) (13). The study suggested that therapeutic modalities aimed at early augmentation of viral clearance (IFN-β, remdesivir), lowering of type-I IFN autoantibody titers (plasmapheresis), and early initiation of corticosteroids upon hypoxemia onset may help improve patient outcomes (13). Another recent study reported that 4 APECED patients with neutralizing autoantibodies to type-I IFNs developed mild COVID-19; all 4 patients were females with ages younger than 26 years old, without underlying autoimmune pneumonitis (29). Currently, therapies for COVID-19 in patients with rare genetic disorders are extrapolated from randomized controlled trials (RCTs) in the general population and published experience is limited. Our two APECED patients had neutralizing autoantibodies directed against IFN-α and IFN-ω and pre-existing lung disease, which both represent risk factors for progression to severe COVID-19. They received bamlanivimab and etesevimab within 4-6 days of their symptom onset and tolerated treatment well without infusion or other adverse reactions. Patient 1, who in addition to APECED and pre-existing lung disease was at risk of severe COVID-19 due to obesity, developed moderate hypoxemia and pulmonary embolism and received methylprednisolone, remdesivir, and anticoagulation in addition to bamlanivimab and etesevimab. Due to his hypoxemic disease, we did not consider IFN-β administration. He fully recovered without the need for ICU admission or mechanical ventilation. Patient 2 had a milder disease course without hypoxemia or pneumonia.

While we cannot ascertain whether mAbs directed to SARS-CoV-2 spike protein are effective in APECED given the small number of treated patients and co-administration of remdesivir and corticosteroids in Patient 1, RCTs have shown SARS-CoV-2 viral load decline, and/or decreased rates of hospitalization and/or mortality when bamlanivimab and etesevimab or other SARS-CoV-2 spike protein-targeted mAbs are started early in the course of COVID-19 (21, 22, 30). Therefore, such mAbs should be considered early in the course of COVID-19 infection in APECED patients, who have a high risk of severe disease and for whom RCTs are not feasible to perform due to the rarity of APECED. Caution should be exercised with administration of these mAbs in hypoxemic COVID-19 patients until more clinical data are available to ascertain their safety. Notably, disseminating the clinical experience of treating COVID-19 in patients with APECED and other inborn errors of immunity who have a high risk of severe disease and for whom RCTs are difficult to perform due to their rarity is important to inform healthcare providers and improve patient outcomes.

## Data Availability Statement

The original contributions presented in the study are included in the article/**Supplementary Material**. Further inquiries can be directed to the corresponding author.

## Ethics Statement

The studies involving human participants were reviewed and approved by the NIH Institutional Review Board (IRB) (11-I-1087). The patients/participants provided their written informed consent to participate in this study. Written informed consent was obtained from the individual(s) for the publication of any potentially identifiable images or data included in this article.

## Author Contributions

ML designed the study. EF, MS, TD, JB, and ML participated in the clinical care to the patients. EF, MS, and SO compiled clinical data and wrote the first draft of the manuscript. LR, ES, PB, JS, and SR performed experiments. EF, MS, SO, SR, LN, SH, and ML analyzed data. ML revised the manuscript. All authors contributed to the article and approved the submitted version.

## Funding

This research was supported by the Division of Intramural Research of the National Institute of Allergy and Infectious Diseases, the National Institute of Dental and Craniofacial Research, and the NIH Clinical Center, NIH.

## Conflict of Interest

The authors declare that the research was conducted in the absence of any commercial or financial relationships that could be construed as a potential conflict of interest.

## Publisher’s Note

All claims expressed in this article are solely those of the authors and do not necessarily represent those of their affiliated organizations, or those of the publisher, the editors and the reviewers. Any product that may be evaluated in this article, or claim that may be made by its manufacturer, is not guaranteed or endorsed by the publisher.
