# Supplementary material for: SARS-CoV-2 Spike Protein-Directed Monoclonal Antibodies May Ameliorate COVID-19 Complications in APECED Patients
Source: Front Immunol. 2021 Aug 24;12:720205. doi: 10.3389/fimmu.2021.720205 (PMC8421855; doi:10.3389/fimmu.2021.720205)
Supplement: Supplementary file 1 [file DataSheet_1.docx]

Supplementary Material

**Supplementary Figure 1**

**
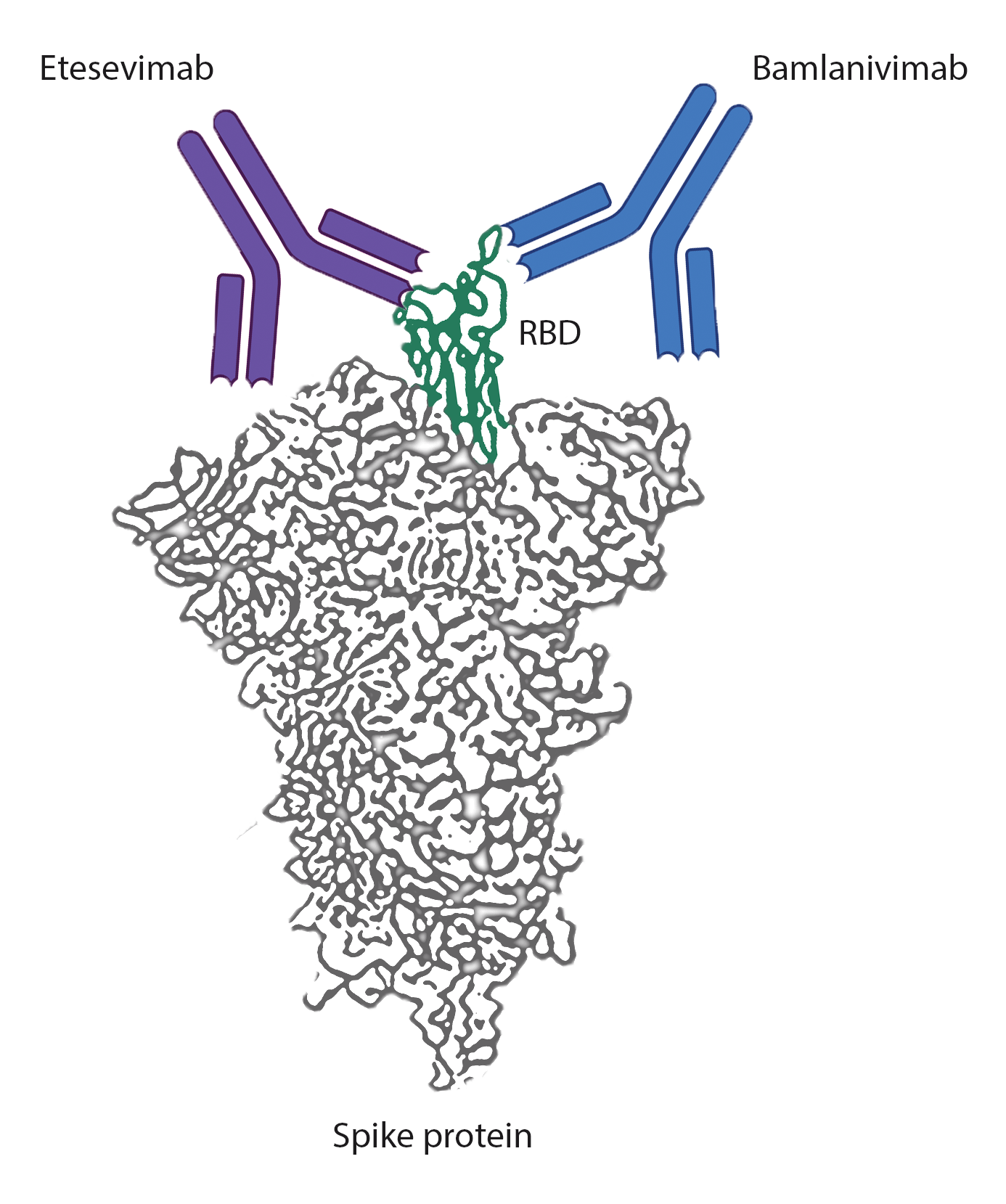
Supplementary Figure 1. Schematic representation of binding of bamlanivimab and etesevimab to the SARS-CoV-2 spike protein epitopes of the receptor binding domain (RBD) in the open conformation.** Created with BioRender.com.

**Supplementary Figure 2**


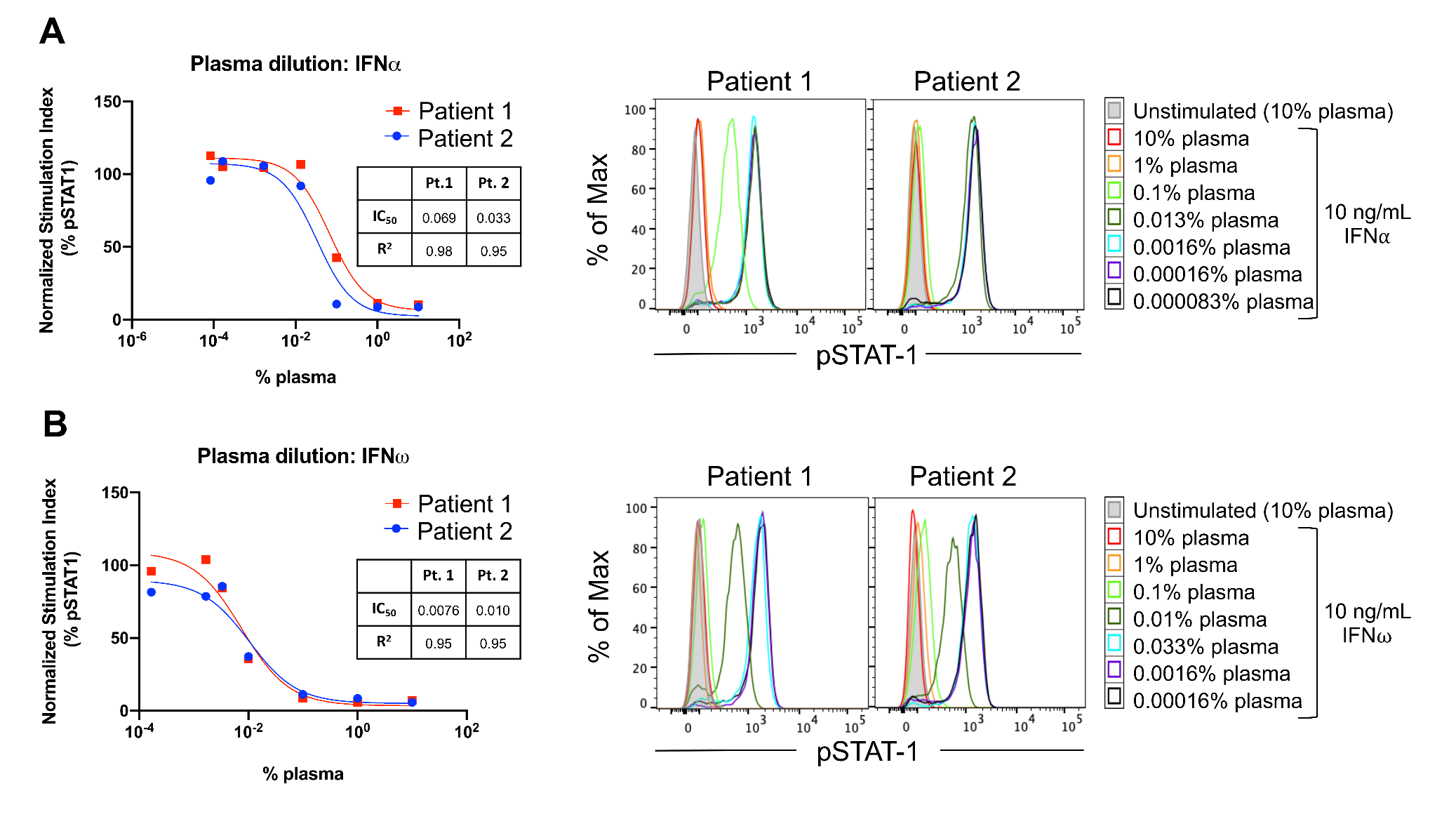
**Supplementary Figure 2. Neutralizing autoantibodies directed against IFN-α and IFN-ω in the APECED patients included in our study.** A, Median inhibitory concentration (IC50) curves representing IFN-α2–induced pSTAT1 levels in healthy donor cells in the presence of serial dilutions of patient plasma. The stimulation index (stimulated over unstimulated condition) for patient plasma was normalized against that of 10% healthy control plasma. Patient 1: IC50 = 0.069%, R2 = 0.98; Patient 2: IC50 = 0.033%, R2 = 0.95. R2, coefficient of determination. B, Median inhibitory concentration (IC50) curves representing IFN-ω–induced pSTAT1 levels in healthy donor cells in the presence of serial dilutions of patient plasma. The stimulation index (stimulated over unstimulated condition) for patient plasma was normalized against that of 10% healthy control plasma. Patient 1: IC50 = 0.0076%, R2 = 0.95; Patient 2: IC50 = 0.010%, R2 = 0.95. IFN, interferon; R2, coefficient of determination.

**Supplementary Figure 3**

**
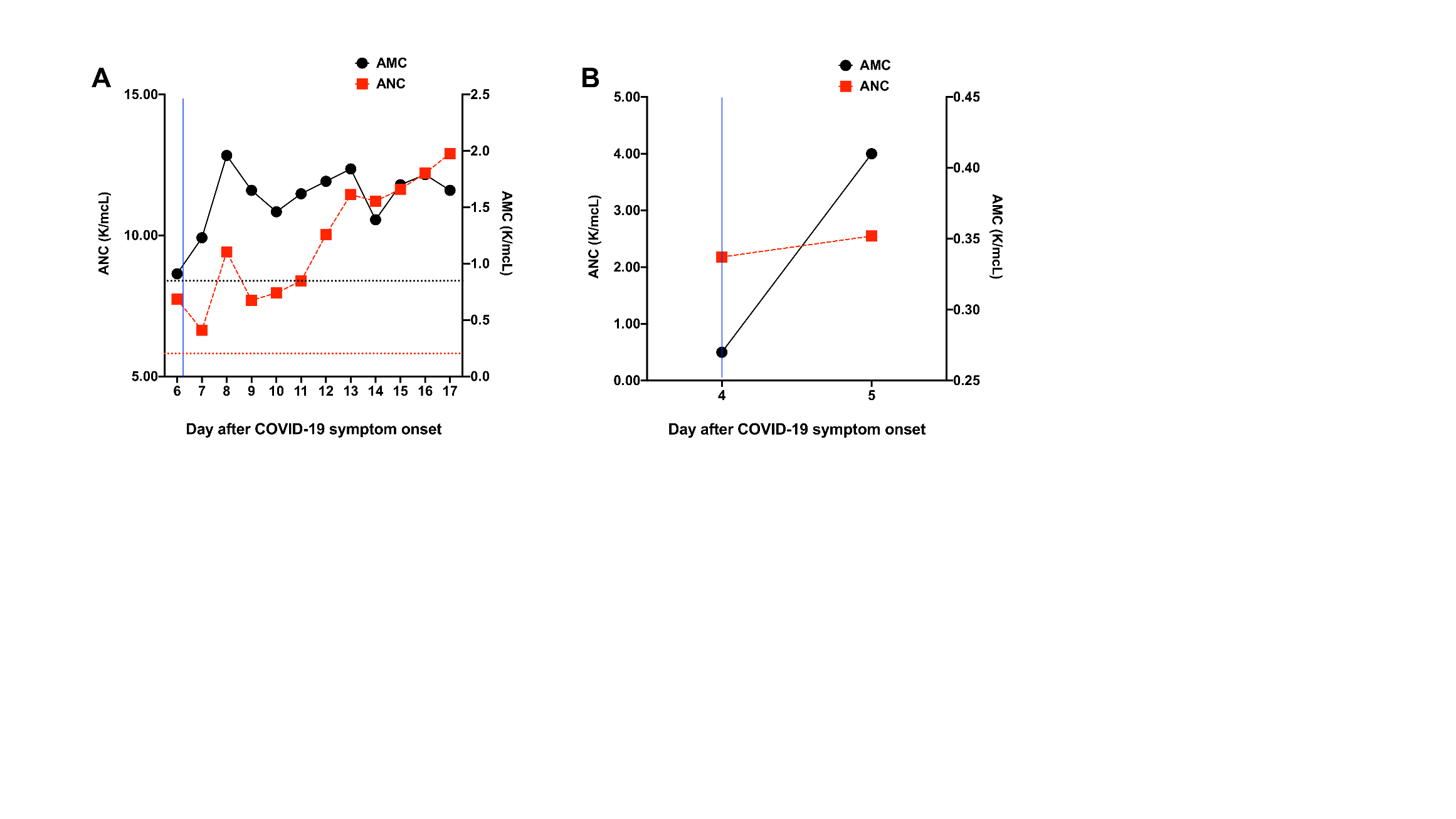
**

**Supplementary Figure 3. Temporal evolution of peripheral blood neutrophil and monocyte counts in the patients included in our study.** A, Temporal evolution of absolute neutrophil count (ANC) and absolute monocyte count (AMC) for Patient 1. The vertical blue line corresponds to the day when the patient was initiated on methylprednisolone, remdesivir, and bamlanivimab and etesevimab. B. Temporal evolution of ANC and AMC for Patient 2. The vertical blue line corresponds to the day of bamlanivimab and etesevimab infusion, ANC/AMC were drawn prior to this infusion. Horizontal dotted red and black lines indicate the upper limit of normal range for ANC (normal range 1.78-5.38 K/mcL) and AMC (normal range 0.30-0.82 K/mcL), respectively.
